# Genome-scale metabolic modeling reveals specific vaginal *Lactobacillus* strains and their metabolites as key inhibitors of *Candida albicans*

**DOI:** 10.1128/spectrum.02984-24

**Published:** 2025-04-16

**Authors:** Tianqi Hu, Ya Meng, Changying Zhao, Dashuang Sheng, Sijie Yang, Junhui Dai, Tiantian Wei, Yiming Zhang, Guoping Zhao, Yanan Liu, Qinghua Wang, Lei Zhang

**Affiliations:** 1 Microbiome-X, School of Public Health, Cheeloo College of Medicine, Shandong University 66555 , Jinan, China; 2 State Key Laboratory of Reproductive Medicine and Offspring Health, Center for Reproductive Medicine, Institute of Women, Children and Reproductive Health, Shandong University 12589, Jinan, China; 3 State Key Laboratory of Microbial Technology, Shandong University, Qingdao, China; 4 Jinan Institute of Child Health Care, Children’s Hospital Affiliated to Shandong University (Jinan Children’s Hospital), Jinan, China; 5 School of Biological Science and Technology, University of Jinan 12413, Jinan, China; Nanchang University, Nanchang, Jiangxi, China

**Keywords:** vulvovaginal candidiasis, *Candida albicans*, *Lactobacillus*, genome-scale metabolic models (GEMs), metabolic interactions

## Abstract

As the predominant constituents of the vaginal microbiome in healthy women, *Lactobacillus* species are considered essential in maintaining a homeostatic vaginal microbiome. Specific *Lactobacillus* species can produce beneficial metabolites to support their persistence within the host environment and inhibit *Candida albicans* colonization. Due to the extensive diversity of *Lactobacillus* species and their metabolites, comprehensively investigating all possible interactions remains challenging. This study employed an integrative approach combining genome-scale metabolic modeling, metagenomic sequencing, and *in vitro* validation to explore *Lactobacillus* and *C. albicans* interactions. Pairwise simulations of 159 *Lactobacillus* strains with *C. albicans* revealed that most strains exhibit inhibitory effects, altering fungal amino acid and carbohydrate metabolism. Key inhibitory metabolites identified included formate, L-lactate, and L-malate. Metagenomic analysis of vaginal swabs from 20 vulvovaginal candidiasis (VVC) patients and 20 healthy women showed a correlation between *Lactobacillus* species abundance and reduced *C. albicans* colonization. *In vitro* experiments confirmed the inhibitory effects of these metabolites and the selected *Lactobacillus* strains on *C. albicans* growth, thereby validating our computational predictions. These findings provide insights into the metabolic interactions within the vaginal microbiome and pave the way for targeted microbial or metabolite-based therapeutic strategies to manage VVC.

**IMPORTANCE:**

Vulvovaginal candidiasis is a prevalent fungal infection with significant implications for women’s health, caused primarily by *Candida albicans*. Although the protective role of a *Lactobacillus*-dominated vaginal microbiome is well established, the metabolic mechanisms underlying the interactions between *Lactobacillus* species and *C. albicans* remain inadequately understood. Specifically, the *Lactobacillus* species that effectively inhibit *C. albicans* and the metabolic pathways involved warrant further investigation. This study offers novel insights into the metabolic mechanisms underlying *Lactobacillus* antagonism against *C. albicans*. By identifying critical metabolic pathways and inhibitory metabolites, this study enhances our understanding of vaginal microbiome dynamics and host-microbe interactions. The findings suggest that key *Lactobacillus* strains and their metabolites could significantly reduce harmful levels of *C. albicans*, paving the way for future therapeutic strategies that leverage these microbial characteristics to promote vaginal health.

## INTRODUCTION

The human vaginal microbiota comprises a diverse array of microorganisms that play a mutualistic role in maintaining vaginal health (1). Disruption of the vaginal microbiota and its metabolic profile can lead to the colonization of various pathogenic microorganisms, such as *Candida albicans*, resulting in infections like vulvovaginal candidiasis (VVC) (2). VVC is one of the most common fungal infections, affecting approximately 75% of women at least once in their lifetime and significantly impacting their quality of life (3). Notably, more than 80% of VVC cases are associated with infections by *C. albicans* (4), an opportunistic pathogen that typically exists in a symbiotic form and shares an ecological niche with bacteria. *C. albicans* attacks vaginal epithelial cells through adhesion factors and exotoxins, triggering an inflammatory response and causing tissue damage, which further contributes to the development and severity of VVC (5). During the progression of VVC, an imbalance in the vaginal microbiota often arises, characterized by a decrease in *Lactobacilli* abundance, alterations in microbial community structure, and significant changes in the composition of vaginal metabolites (6). Correlative studies in metagenomics have revealed an association between VVC and variations in the vaginal microbiome, demonstrating increased microbial diversity in the vaginal microbiota of patients with VVC, along with a decrease in beneficial *Lactobacillus* species such as *Lactobacillus crispatus* (7).

*Lactobacillus* is the major component of the healthy vaginal microbiota in women (8). Recent studies have found links between *Lactobacillus* and a range of health benefits that are critical for maintaining reproductive health. For instance, a *Lactobacillus*-dominated microbiota correlates with a marginal presence of fungal taxa (9), thereby limiting the potential for opportunistic infections. The lactate produced by *Lactobacillus* contributes to the acidification of the vaginal environment, acting as a natural barrier against the proliferation of various pathogens (10). The small molecule (11) produced by *Lactobacillus* can inhibit the transition of *C. albican*s from yeast to hyphal morphology, which is considered a hallmark virulence factor of VVC (12). Furthermore, *Lactobacillus* species can inhibit the adhesion and invasion of *Candida* species into vaginal epithelial cells, thereby mitigating the risk of infections (13). The colonization and sustained dominance of *Lactobacillus* are essential traits of a healthy vaginal microbiota, predominantly represented by species such as *L. crispatus*, *Lactobacillus gasseri*, *Lactobacillus iners*, and *Lactobacillus jensenii* (14). Multiple studies have demonstrated that the protective effects of *Lactobacillus* against sexually transmitted infections, bacterial vaginosis, and VVC are intrinsically linked to their ability to produce organic acids and secretory antimicrobial compounds, which are crucial in maintaining the ecological balance of the vaginal microbiota and preventing dysbiosis (15–17). These characterizations position *Lactobacillus* and their metabolites as potential therapeutic options for VVC (18). Previous studies indicate that colonization with *Lactobacillus* can antagonize *C. albicans*; for example, *L. rhamnosus*-derived metabolites have been reported to antagonize *C. albicans* (19).

However, the specific *Lactobacillus* species that effectively inhibit *C. albicans*, as well as the metabolic mechanisms involved in this process, require further exploration. Given the wide variety of *Lactobacillus* and their metabolites in the vagina, it is challenging to allocate significant resources in experimental verification for all potential interactions. A promising method to explore the mechanism of metabolic interactions between *C. albicans* and *Lactobacillus* is through genome-scale metabolic models (GEMs). GEMs provide mathematical representations of biological organism metabolic networks. By establishing gene-protein-reaction (GPR) associations using genomic annotation information and experimental data, GEMs enable the simulation of the entire metabolic flux of organisms under predefined environmental conditions (20). Recent studies have developed high-quality GEMs for the human microbiota, advancing research on microbial interactions and their pairwise relationships (21). To obtain deeper insights into the metabolic mechanism between *Lactobacillus* and *C. albicans* and to identify key *Lactobacillus* species and metabolites, we constructed paired growth models of *Lactobacillus* and *C. albicans*. Based on these models, we computationally analyzed their interactions and metabolic functions and validated the predicted effects of inhibitory *Lactobacillus* and their metabolites on inhibiting *C. albicans* through *in vitro* experiments. This approach elucidated the mechanisms of metabolic interactions between *Lactobacillus* and *C. albicans*, providing novel insights for further research in this field.

## RESULTS

### Improvements of *C. albicans* GEM

**Fig 1:**
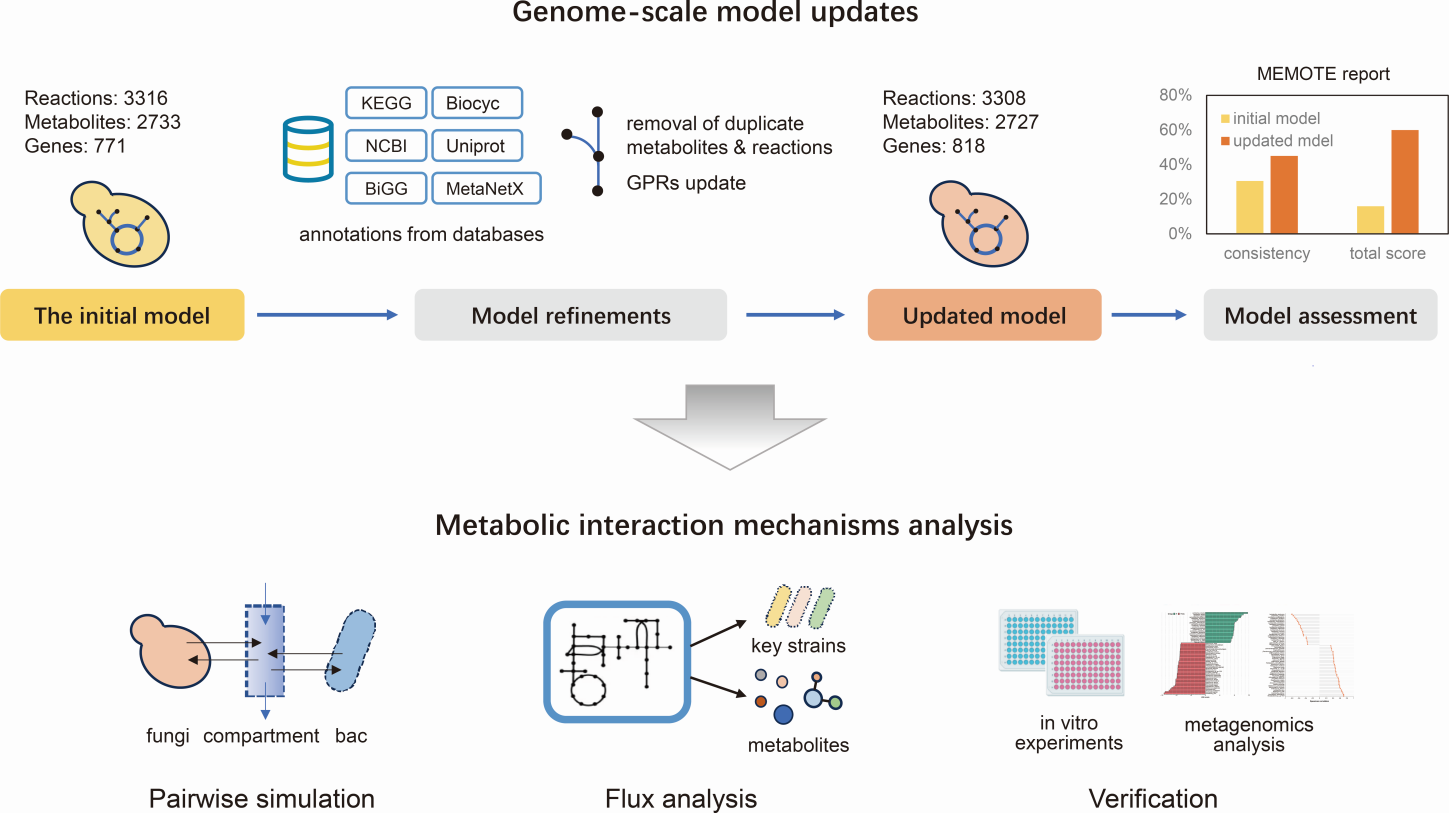
Overview of model update steps and metabolic interaction analysis.

We adopted an existing *C. albicans* genome-scale metabolic model (BioModels ID 2110210002) as our starting point (22). The initial model, reconstructed and curated using phenotypic microarray data and by rectifying erroneous energy-generating cycles, comprised 771 genes, 3,316 metabolic reactions (including 3,082 network reactions and 234 exchange reactions), and 2,733 metabolites. We systematically improved the *C. albicans* GEM through several rounds of updates (Fig. 1).

We eliminated duplicate reactions and metabolites, resulting in a reduction of the model by seven reactions and six metabolites. Additional genes from MODEL2109130014 (23) and iRV781 (24) were integrated to enhance genome coverage. This integration, combined with updated annotations from Kyoto Encyclopedia of Genes and Genomes (KEGG) (25), UniProt (26), and BioCyc (27), resulted in the addition of 70 new GPRs, modification of 1 GPR, and removal of 3 incorrect GPRs. Reaction and metabolite annotations were curated using data from five databases (cf. “Materials and Methods”). Additionally, each reaction was assigned to a single explicit subsystem based on KEGG and BioCyc annotations to facilitate pathway analysis. These updates significantly improved the model’s reliability and functional relevance.

The compatibility of our model was checked with metabolic modeling standards by running MEMOTE tests (28). The overall MEMOTE score of the updated model increased by 44% compared to that of the initial model (File S1).

### *In silico* prediction of *Lactobacillus-C. albican*s interactions

Using 159 publicly available GEMs for *Lactobacillus* strains (21), we conducted pairwise metabolic analysis by integrating our *C. albicans* model with each *Lactobacillus* GEM (29). Growth simulations were performed using compositions of vaginal culture media that closely mimic the typical vaginal environment (30).

**Fig 2:**
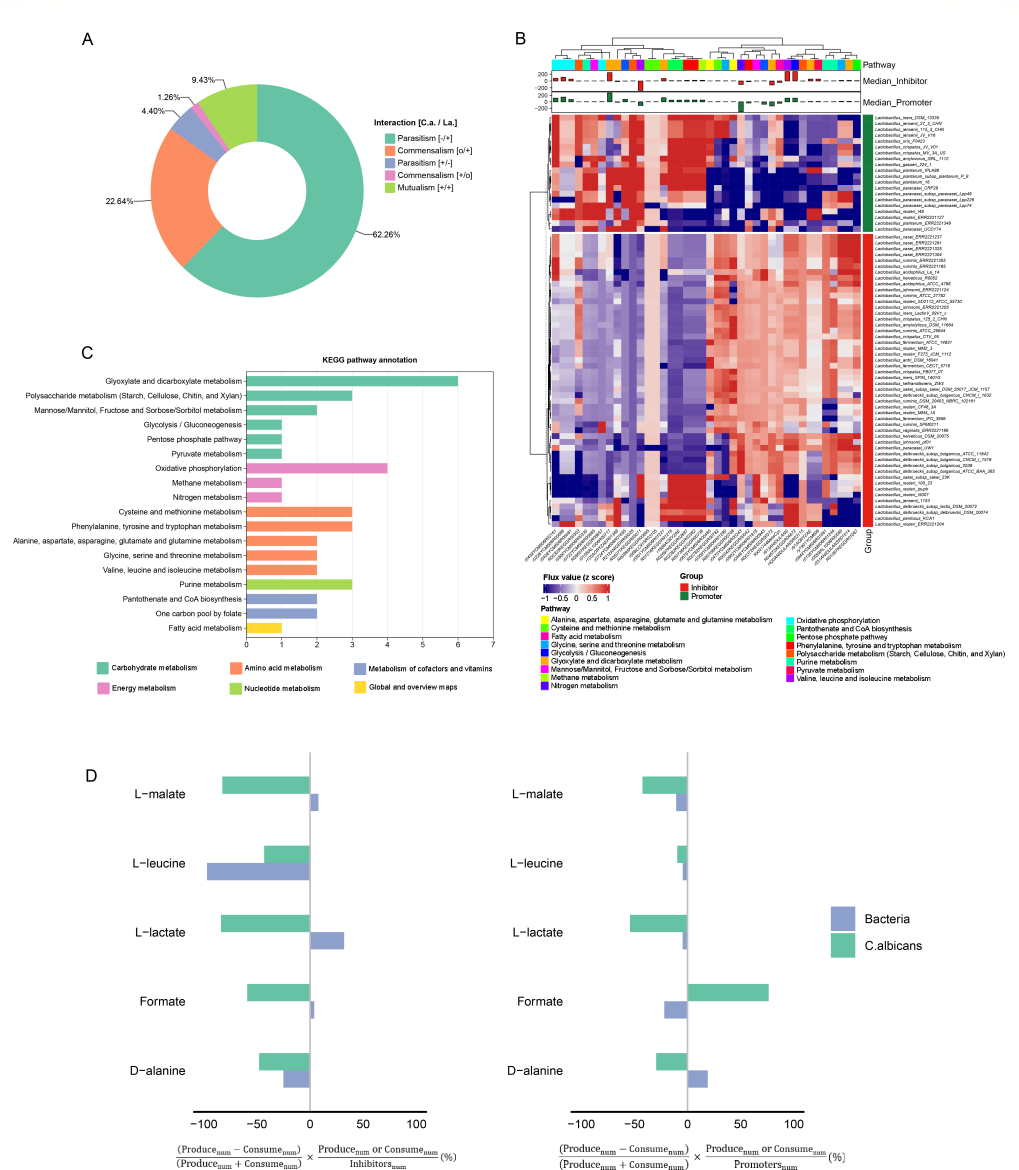
Pairwise *in silico* interaction experiments. (A) Distribution of interaction type for *C. albicans* (C.a.) and *Lactobacillus* species (La.). Interactions have positive (+), negative (−), or no (**O**) effects on growth rates of *C. albicans* or *Lactobacillus* as indicated for interaction types. (B) Metabolic reactions of *C. albicans* with the most substantially differing flux rates of *C. albicans* when paired with the top 50 inhibiting or top 20 promoting *Lactobacillus*. Top: median *C. albicans* flux differences across inhibiting or promoting *Lactobacillus* paired with *C. albicans*. (C) KEGG pathway annotation of metabolic pathways. (D) Analysis for selected metabolites based on exchange reaction fluxes of simulated fungal–bacterial pairs for 99 inhibiting or 24 promoting bacterial species (cf. Table S2). *x* > 0 for produce and *x* < 0 for consume.

We identified the interaction type of each *C. albicans-Lactobacillus* pair by analyzing the differences in predicted pairwise growth rates compared to growth rates derived from individual simulations using a flux balance analysis (FBA) approach (31) (methods, paired simulations; Table S1). Our analysis indicated that the predominant interaction types based on the predicted growth rate differences were parasitism (here, negative effect on *C. albicans* growth, positive growth effect on *Lactobacillus*) and commensalism (indicating no growth effect on *C. albicans* while exerting a positive effect on *Lactobacillus*), which collectively accounted for over 84% of the observed interactions (Fig. 2A). Notably, the majority of *Lactobacillus* strains (62.3%), such as *Lactobacillus crispatus* CTV-05 and *Lactobacillus johnsonii* ATCC33200, demonstrated an inhibitory effect on the growth of *C. albicans* in paired simulations. Only a limited number of instances of parasitism (here, negative effect on *Lactobacillus* growth and positive growth effect on *C. albicans*), commensalism (indicating no growth effect on *Lactobacillus*, positive effect on *C. albicans*), and mutualism (indicating positive growth effect for both *C. albicans* and *Lactobacillus*) were observed.

### Metabolic pathway analysis of *C. albicans* responses to *Lactobacillus*

To analyze the specific metabolic pathways influencing the growth of *C. albicans*, we investigated reaction fluxes particular to *C. albicans* derived from both its individual growth and paired simulations with *C. albicans–Lactobacillus* models. The reactions of *C. albicans* were selected based on the most significant flux differences between the flux value of the response for individual growth of *C. albicans* and the median flux value of the response for *C. albicans* paired with predicted growth-inhibiting bacterial GEMs (Fig. S1A). The metabolic pathway analysis indicated that inhibiting *Lactobacillus* strains primarily affects the amino acid and carbohydrate metabolism of *C. albicans* (Fig. S1B). Specifically, these alterations were particularly present in pathways related to glyoxylate and dicarboxylate metabolism, glycolysis, valine, leucine, and phenylalanine. We subsequently analyzed the *C. albicans*-specific reaction fluxes of *C. albicans* paired with the top 50 inhibiting *Lactobacillus* strains and the top 25 promoting *Lactobacillus* strains, selected based on their predicted influence on *C. albicans* growth rates. This ensured that the differences between paired *C. albicans–Lactobacillus* and individual model simulations were most pronounced (Fig. 2B). Altered reaction fluxes were observed across major pathways, including carbon, amino acid, and purine metabolism, indicating that *C. albicans* growth was influenced by the modulation of carbohydrate metabolism and the availability of amino acids. *Lactobacillus* was predicted to influence *C. albicans* growth by competing for resources through carbohydrate and amino acid metabolism (Fig. 2C). For example, we predict decreased reaction fluxes that consumed L-tyrosine in *C. albicans* when paired with growth-inhibiting *Lactobacillus*, since the flux data related to L-tyrosine metabolism showed a decrease compared to the flux observed during the individual growth of *C. albicans* and its pairing with growth-promoting *Lactobacillus*.

### Identification of key metabolites in *Lactobacillus-C. albicans* interactions

Subsequently, we investigated the fluxes of metabolite exchange reactions to identify potential metabolites that influence the growth rate of *C. albicans*. In paired *in silico* models, exchange reactions enable metabolites to shuttle between the joint compartments. These joint compartments serve as connections between the respective *C. albicans* model and the *Lactobacillus* model, enabling the prediction of potential metabolites that influence the growth rate of *C. albicans*. This includes substances produced by *Lactobacillus* that inhibit the growth of *C. albicans* and substrates for which *Lactobacillus* competes with *C. albicans*. We analyzed the metabolites produced or consumed by *Lactobacillus* or *C. albicans* using the flux of metabolites during the pairing of *C. albicans* with inhibiting or promoting *Lactobacillus* (Table S2). Many amino acids, such as L-leucine and D-alanine, along with other factors like formate and L-lactate, are predicted to be differentially consumed or produced by fungi or bacteria (Fig. 2D). We found that both *C. albicans* and *Lactobacillus* consume L-leucine for growth. The utilization of D-alanine differs between inhibiting *Lactobacillus* and promoting *Lactobacillus* in their pairing with *C. albicans*. The inhibiting *Lactobacillus* tends to consume D-alanine, while the promoting *Lactobacillus* tends to produce D-alanine. In our simulation of *Lactobacillus* pairing with *C. albicans*, we observed that inhibiting *Lactobacillus* was more likely to produce organic acids such as formate, L-lactate, and L-malate compared to promoting *Lactobacillus*. We investigated the metabolic pathways of these organic acids in the inhibitory *Lactobacilli* GEMs and found that *Lactobacillus* could convert glucose into pyruvate via the glycolytic pathway. Pyruvate can then be further reduced to L-lactate acid under the catalysis of L-lactate dehydrogenase or converted into formate through a reaction with coenzyme A, catalyzed by pyruvate formate-lyase. L-malate is primarily produced through the reaction of fumarate, an intermediate in the tricarboxylic acid (TCA) cycle, with water, catalyzed by fumarase. This process helps maintain cellular energy balance and may indirectly inhibit *C. albicans* growth by altering its growth environment.

### *In vitro* experiments and metagenomics analysis support *Lactobacillus-C. albicans* metabolic interactions

**Fig 3:**
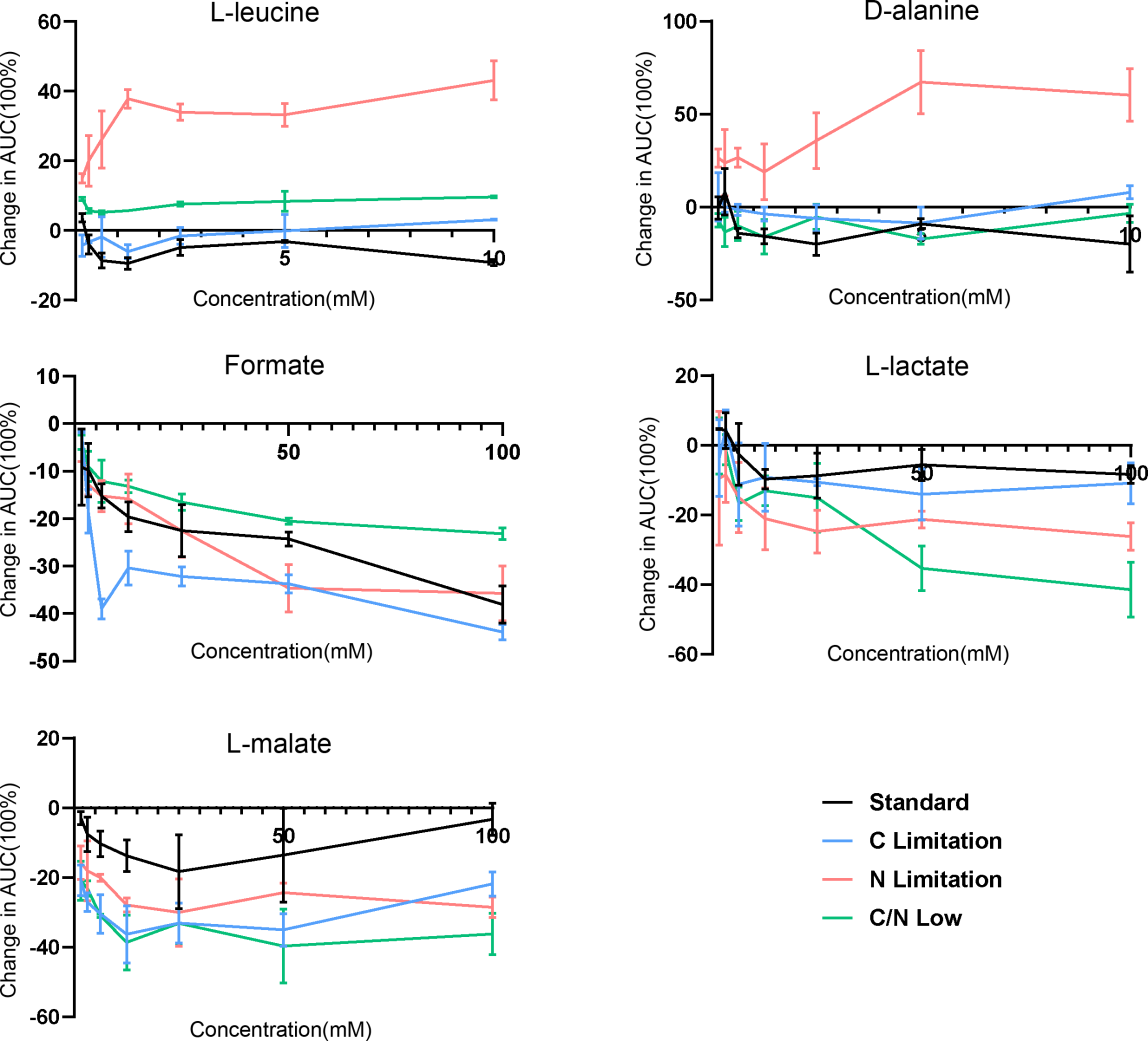
Metabolite experiment supporting *in silico* predictions. Area under the curve (AUC) measurements for *C. albicans* growth in the presence of selected metabolites in a series of concentration dilution experiments. AUCs were determined for three replicates. Mean change compared to medium-only controls is shown with SDs as error bars.

To validate our *in silico* analysis, we examined the growth of *C. albicans* in the presence of various metabolites, conducted metagenomic sequencing on samples from 40 individuals, and evaluated *C. albicans* growth in *Lactobacillus* cell-free supernatant (CFS). We focused on five metabolites that were predominantly utilized or produced by fungi or bacteria in our paired metabolic simulations (Fig. 2D). In our experiments, *C. albicans* was cultured with these metabolites to assess their effects on growth promotion or inhibition under varying carbon and nitrogen availability. We observed moderate inhibitory effects from formate, L-lactate, and L-malate, which are predicted to be produced by *Lactobacillus* when paired with growth-inhibiting strains of *C. albicans*. Conversely, L-leucine and D-alanine exhibited concentration-dependent growth-promoting effects on *C. albicans*, particularly under conditions of nitrogen limitation (Fig. 3).

**Fig 4:**
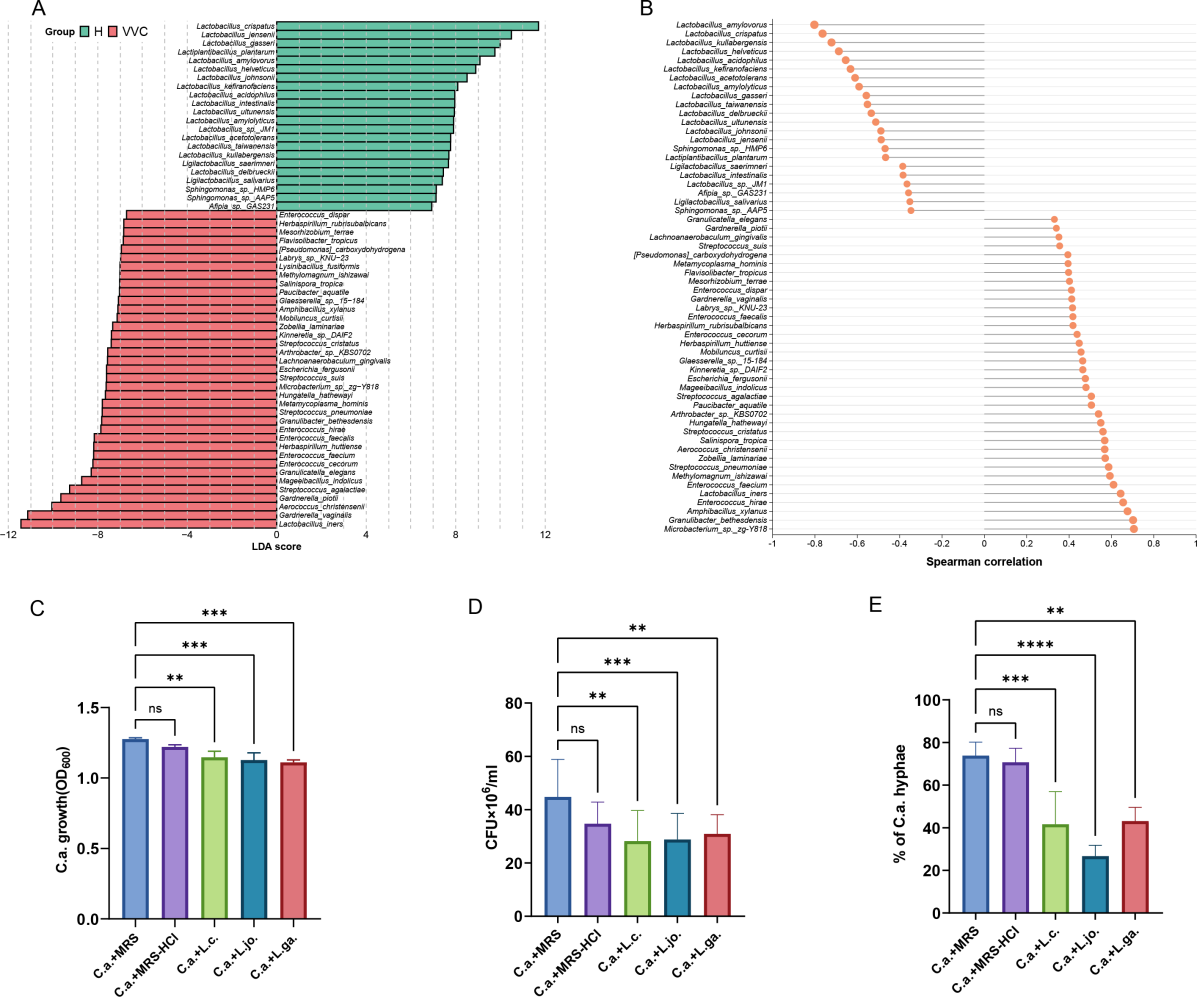
Clinical and *Lactobacillus* CFS experimental data supporting *in silico* predictions. (A) The difference of species-level relative abundance in the VVC group and healthy group by LEfSe (*P* < 0.01 and LDA > 2). (B) Correlation between the differential microorganisms identified through LEfSe analysis and *C. albicans*. (C) Effects of the CFS and acidified De Man, Rogosa, and Sharpe medium (MRS; MRS-HCl) on *C. albicans* growth compared to *C. albicans* grown either in MRS (C.a.). The graph shows the OD600 mean ± SD of triplicate samples from five different experiments. **, *P* < 0.01; ***, *P* < 0.001; ns, no significance. (D) The viable fungal cells were counted by a hemocytometer after 24 hours of contact with the different CFS, MRS, or MRS-HCl. Trypan blue staining allowed the exclusion nonviable cells. The graph reports the mean CFU ± SD from five different experiments. **, *P* < 0.01; ***, *P* < 0.001; ns, no significance. (E) CFS effect on *C. albicans* hyphal formation. Data in the graph are the mean % ±SD of four analyzed fields for each condition. The experiments were repeated three times. **, *P* < 0.01; ***, *P* < 0.001; ****, *P* < 0.0001; ns, no significance.

To explore whether metabolic interactions primarily drive the abundance-based association between *Lactobacillus* and *C. albicans*, we analyzed the metagenomic sequencing results of vaginal swabs from healthy women and VVC women. A total of 59 microorganisms at the species level exhibited significant differences between the VVC group and the healthy group by using linear discriminant analysis (LDA) effect size (LEfSe) analysis (*P* < 0.01, Wilcoxon rank-sum test; LDA > 2.0). The LEfSe results demonstrated that compared to the VVC group, the relative abundance of *Lactobacillus* in the healthy group was significantly increased, particularly for species such as *L. crispatus*, *L. jensenii*, *L. gasseri*, *Lactobacillus amylovorus*, and *L. johnsonii*. In contrast, the relative abundance of *L. iners*, *Gardnerella vaginalis*, *Aerococcus christensenii*, *Gardnerella piotii*, *Streptococcus agalactiae*, and other pathogenic microorganisms was enriched in the vaginal microbiome of women in the VVC group (Fig. 4A). Subsequently, we investigated the correlation between the differential microorganisms that were identified through LEfSe analysis and *C. albicans*. We found that *Lactobacillus* species, including *L. amylovorus* (ρ  = −0.80), *L. crispatus* (ρ  = −0.76), *Lactobacillus kullabergensis* (ρ  = −0.72), *Lactobacillus helveticus* (ρ  = −0.69), *L. gasseri* (ρ  = −0.56), and *L. johnsonii* (ρ  = −0.49) were negatively correlated with *C. albicans* (Fig. 4B).

Among the species selected for *in vitro* experiments, *L. crispatus*, *L. johnsonii*, and *L. gasseri* exhibited inhibitory effects based on *in silico* predictions (Table S1). These species were also found to be significantly negatively correlated with *C. albicans* abundance in the vagina. We measured the average pH of the CFS from this *Lactobacillus* as 4.8, while the pH of the De Man, Rogosa, and Sharpe medium (MRS) was 6.0. The acidified MRS (MRS-HCl) exhibited a similar average pH of 4.8, comparable to that of the CFS. We assessed the impact of CFS obtained from three different *Lactobacillus* species on the virulence traits of *C. albicans*, specifically growth, viability, and hyphal formation. Our results indicate that in the presence of MRS-HCl, the growth of *C. albicans* was partially reduced compared to the control sample (*C. albicans* grown in non-acidified MRS), although this difference did not reach statistical significance. In contrast, the incubation of *C. albicans* with CFS resulted in a significant reduction in growth compared to the *C. albicans* grown in MRS (Fig. 4C). Subsequently, we investigated whether this growth inhibition was accompanied by a decrease in viability. It was observed that after co-incubation of *C. albicans* with *Lactobacillus* CFS for 24 hours, there was a significant reduction in viable cells (Fig. 4D). By evaluating the ability of different CFS to inhibit hyphal formation in *C. albicans*, we found that all CFS significantly inhibited *C. albicans* hyphal formation (Fig. 4E).

## DISCUSSION

This study demonstrates the utility of GEMs in elucidating the complex metabolic interactions between *Lactobacillus* strains and *C. albicans* within the vaginal environment. By integrating computational predictions with metagenomic sequencing data and *in vitro* validation, we identified specific *Lactobacillus* strains and their metabolites that exert inhibitory effects on the growth of *C. albicans*.

We extended the concept of using *in silico* metabolic interaction calculations to predict paired beneficial or harmful effects on coexisting organisms (32, 33) to predictions of *Lactobacillus-C. albicans* interactions. We improved an existing GEM of *C. albicans*, which resulted in an enhancement in consistency and a higher MEMOTE total score. Our pairwise simulations of *C. albicans* and 159 *Lactobacillus* strains revealed that the majority of *Lactobacillus* species, particularly *L. crispatus*, *L. gasseri*, and *L. johnsonii*, exhibit inhibitory effects on the growth of *C. albicans*. This finding underscores the crucial role of these *Lactobacillus* species in maintaining vaginal health. Notably, the interaction types predominantly observed were parasitism and commensalism. These interactions are likely attributable to the ability of *Lactobacillus* to produce metabolites, such as lactic acid, which inhibit the growth of *C. albicans* while conferring a competitive advantage to *Lactobacillus* (34, 35). Only a small subset of the simulations indicated mutualistic interactions, suggesting that the metabolic balance between *C. albicans* and *Lactobacillus* is often tipped in favor of bacterial inhibition of fungal growth.

The metabolic pathway analysis further clarified the mechanisms by which *Lactobacillus* strains inhibit *C. albicans*. We identified significant alterations in the amino acid and carbohydrate metabolism pathways of *C. albicans* when paired with inhibitory *Lactobacillus* strains. Specifically, pathways related to glyoxylate and dicarboxylate metabolism, as well as glycolysis and amino acid biosynthesis (e.g., valine, leucine, and phenylalanine), were notably affected. The growth of *C. albicans* is influenced by the regulation of carbohydrate metabolism (36) and the availability of amino acids, such as leucine (37), tryptophan, or phenylalanine (38). Predicting significant differences in reaction flux among top promoters and inhibitors may serve as potential targets for identifying antifungal agents (39). *Lactobacillus* species can shape the vaginal environment by consuming and releasing metabolites (40), and the new environment forces *C. albicans* to adjust its metabolic pathways, which might result in the loss of certain characteristics and a reduction in *C. albicans* infectivity. As the human vaginal epithelium is glycogen rich, vaginal *lactobacilli* likely derive the majority of their carbon and energy through the fermentation of glycogen, converting it ultimately into short-chain fatty acids and organic acids (41). Our analysis of exchange reactions revealed that key metabolites, such as formate, L-lactate, and L-malate, were produced in higher quantities by inhibitory *Lactobacillus* strains. These metabolites likely play a direct role in suppressing *C. albicans* growth, as demonstrated by our *in vitro* experiments. Additionally, we found that both inhibitory and growth-promoting bacteria consumed L-leucine, with inhibitory bacteria showing higher consumption rates. Alanine utilization differed between inhibitory and growth-promoting bacteria, with inhibitory strains tending to consume alanine while growth-promoting strains tended to produce it. These patterns suggest potential competitive mechanisms, whereby *Lactobacillus* may deprive *C. albicans* of essential nutrients. Through the analysis of the metabolic reactions of formate, L-lactate, and L-malate in the *Lactobacilli* GEMs, we found that L-lactate and formate can be produced through the conversion of pyruvate. This not only contributes to maintaining glycolysis under anaerobic conditions but also inhibits the growth of pathogens by acidifying the environment. In terms of carbon flux distribution, pyruvate, as a key metabolic intermediate, is regulated by enzyme activity and substrate competition, determining whether it is directed toward the production of L-lactate or formate (42). Additionally, within the TCA cycle, fumarate metabolism is dynamically modulated in response to the cell’s energy status and biosynthetic demands, thereby influencing L-malate production.

Selected metabolite experiments and shotgun metagenomics sequencing support our *in silico* modeling concept based on paired metabolic interaction simulations. Guided by our pairwise simulations, we investigated specific metabolites with growth-promoting or inhibitory effects on *C. albicans* under defined media conditions with carbon and nitrogen limitations. We also found that formate, L-lactate, and L-malate can inhibit *C. albicans* growth. In contrast, L-leucine and D-alanine exhibited concentration-dependent growth-promoting effects on *C. albicans* specifically under nitrogen limitation. These findings indicate that *Lactobacillus* colonization might change nutrient availability, leading to the depletion of nitrogen sources such as amino acids and the accumulation of secondary carbon sources such as lactic or malic acid (19). The utilization of different carbon sources can greatly affect the adaptability and pathogenicity of *C. albicans* (43). These findings collectively underscore the complex metabolic interplay between *Lactobacillus* and *C. albicans*, highlighting potential mechanisms of fungal growth inhibition through nutrient competition and the production of inhibitory metabolites. The metagenomic data from vaginal swabs of healthy women and VVC patients provided additional support for our findings. We observed a significant increase in the relative abundance of beneficial *Lactobacillus* species, such as *L. crispatus*, *L. jensenii*, and *L. gasseri*, in healthy women, whereas women with VVC exhibited a decrease in these species alongside an increase in pathogenic taxa. This inverse relationship between *Lactobacillus* abundance and *C. albicans* dominance highlights the protective role of *Lactobacillus* in maintaining vaginal homeostasis and preventing fungal overgrowth (44, 45).

To further substantiate the idea that the metabolic interactions with *Lactobacillus* could significantly influence the colonization levels of *C. albicans*, we selected the supernatant of *Lactobacillus* strains that exhibited significant inhibitory effects and were isolated from healthy women for our experiments. We assessed the effects of CFS obtained from *L. crispatus*, *L. johnsonii*, and *L. gasseri* strains on *C. albicans*. Our results indicated that the CFS from these *Lactobacillus* species inhibited the growth, activity, and hyphal formation of *C. albicans*. This may be attributed to the presence of substances such as L-lactate and L-malate in the CFS of the *Lactobacillu*s (7, 46).

Our study offers distinctive insights into the mechanisms by which these beneficial bacteria may inhibit fungal growth and virulence. However, our study has some limitations, as the FBA algorithm cannot be used to predict metabolite concentrations since it does not take into account kinetic parameters, and second, regulatory roles, such as enzyme activation, gene expression regulation, etc., are not included in the FBA algorithm. In subsequent studies, GEM can be combined with experimental data or omics data by DynamicME (47), MOOMIN (48), and other methods to more accurately simulate the metabolic process of organisms. Furthermore, although pairwise interactions have been shown to be key drivers of microbial community dynamics (49), given the complexity of microbial networks, pairwise interactions may be influenced by the presence of other species within the system. The construction of community metabolic models and computer simulations of higher-order interactions such as the HiOrCo algorithm (50) need to be resolved to potentially expand our understanding of the intricate relationship between *C. albicans* and the vaginal microbiota.

In conclusion, our computerized analysis of metabolite exchange between *Lactobacillus* and *C. albicans*, complemented by experimental and metagenomic data, establishes a robust framework for predicting and validating potential fungal growth-regulating metabolites. Our strategy of using a computerized metabolism-driven approach to study the *C. albicans-Lactobacillus* relationship in the vaginal environment has yielded promising results. Our study contributes to a deeper understanding of bacterial-fungal interactions and provides new insights into the mechanisms of metabolite exchange between microorganisms. By incorporating metabolic considerations into computational analysis, we have opened new avenues for systems biology and systems medicine research focused on fungal infections and their impact on human health.

## MATERIALS AND METHODS

### Model improvements

The *C. albicans* GEM was generated using a previously published model (22) as a template. This template model was reconstructed and curated using phenotypic microarray data and rectifying erroneous energy-generating cycles. We systematically refined the template model through multiple updates, including annotation curation, GPR updates, and subsystem annotation.

Metabolite formulas and annotations were updated based on KEGG (25) and ChEBI (51) databases. The template model initially contained a total of 2,733 metabolites, of which 2,355 metabolites were annotated with KEGG IDs and 90 with ChEBI IDs. Using the original KEGG IDs (or ChEBI IDs), we obtain the full names, chemical formulas, and corresponding ChEBI IDs (or KEGG IDs) for each metabolite. By comparing old and new information in ChEBI IDs, KEGG IDs, full names, and chemical formulas, respectively, for each metabolite, duplicate metabolites with multiple synonyms and database IDs were identified and removed. The affected reactions were refined accordingly (see Table S3). Based on the correct KEGG IDs (or ChEBI IDs) information, we obtained further information such as PubChem ID (52), BiGG ID (53), and MetaNetX ID (54).

Next, we add new GPRs and genes by merging the template model with MODEL2109130014 (23) and iRV781 (24). Besides, 2,657 reactions were referenced with KEGG (25) and Biocyc (27) databases to verify and update the reaction information including reaction names, equations, and Enzyme Commission (EC) numbers.

Detailed information on the primary databases used for model curation is provided in Tables S3 and S4.

### Pairwise simulations

By reviewing the literature (55, 56), we identified 27 *Lactobacillus* species commonly found in the vaginal microenvironment (Table S5). Based on these species, we selected corresponding *Lactobacillus* GEMs from AGORA2. A total of 159 strain-specific GEMs were used for subsequent pairwise simulations. These GEMs are publicly available and can be downloaded from https://www.vmh.life/files/reconstructions/AGORA2/ (21). Pairwise simulations were adapted from the research conducted by Mohammad H. Mirhakkak and Sascha Schäuble (22). Metabolic modeling simulations require environmental conditions such as media and carbon source availability. To simulate the vaginal environment, we formulated a “general vaginal medium” (Table S6) by referring to the metabolites commonly found in the female vaginal metabolome measured by William F Kindschuh et al. (30) and combining it with the minimal simulated medium detected by the Cobrapy package that ensures the growth of all *Lactobacilli*. Considering that intracellular metabolite concentrations typically range from 1 nM to 10 mM, we adjusted the metabolite concentration in the culture conditions to 1 mM (57) to better mimic physiological conditions. The interaction type between each pair of *C. albicans* and *Lactobacillus* was determined by using FBA to predict the differences in growth rates between individual and paired growth of *C. albicans* and *Lactobacillus*. Specifically, the GEMs of *C. albicans* and paired *Lactobacillus* are connected through a separate compartment (U) that simulates the vaginal environment. The compartment is extracellular and serves as an entry point for nutrients in the simulated vaginal environment, facilitating metabolite exchange between the microorganisms and providing an outlet for final products. By maximizing the biomass objective function of *C. albicans* and paired *Lactobacillus*, it is possible to simulate the growth rate of the two species when they coexist. The objective function for the paired model is defined as a linear combination of the biomass generation reactions for two organisms:


$$maximize\;Z=v_{Candida\;biomass}+v_{Bacteria\;biomass}$$

where $v_{Candidabiomass}$ and $v_{Bacteriabiomass}$ represent the biomass generation rates of *C. albicans* and *Lactobacillus*, respectively. A linear programming-based optimization algorithm is employed to solve the model, aiming to maximize the biomass generation rates of both species simultaneously. Additionally, we further apply parsimonious FBA to minimize the total flux in the metabolic network, ensuring the biological relevance and robustness of the model. The type of interaction between the species was determined by considering the ratio of the growth rate of the co-culture to that of the species alone, as calculated in the following formula:


$$Y_{La\to CA}=log_{2}\left(\frac{G_{CA,pair}}{G_{CA,indv}}\right),Y_{CA\to La}=log_{2}\left(\frac{G_{La,pair}}{G_{La,indv}}\right).$$

Y*_La→CA_* represents the effect of *Lactobacillus* on *C. albicans* growth, while Y*_CA→La_* represents the effect of *C. albicans* on *Lactobacillus* growth. The model-predicted growth rates of *C. albicans* and *Lactobacillus* under paired and individual growth conditions are represented by G*_CA,pair_*, G*_CA,indv_*, G*_La,pair_*, and G*_La,indv_*, respectively (Supplementary material). The Python code and metabolic models for simulating pairwise GEMs are available at https://github.com/Hu-tianqi/Candida-albicans-Lactobacillus--interaction.

### Growth of *C. albicans* in the presence of metabolites

To assess the impact of the metabolites on *C. albicans* growth, *C. albicans* SC5314 was cultured overnight at 30°C in Yeast Extract Peptone Dextrose Medium (YPD, Hopebio, China). The temperature of 30°C was chosen to prevent hyphal formation, which can occur at higher temperatures and interfere with growth curve measurements. After culturing, yeast cells were washed three times with sterile H_2_O by centrifugation for 5 min at 4,200 × *g*. The testing medium was formulated of 1 × yeast nitrogen base (YNB, Solarbio) with either (standard) 0.25% (NH_4_)_2_SO_4_/2% glucose, (C limited) 0.25% (NH_4_)_2_SO_4_/0.25% glucose (N limited), 0.008% (NH_4_)_2_SO_4_/2% glucose, or (C/N low) 0.016% (NH_4_)_2_SO_4_/0.5% glucose. Test substances were obtained from MACKLIN and dissolved in H_2_O at the following concentrations: sodium formate (1 M), sodium L-malate (1 M), sodium L-lactate (1 M), L-leucine (100  mM), and D-alanine (100  mM). Assays were conducted in 96-well plates (TPP, flat bottom) using a 1:2 dilution series and were composed of 180 µL test medium, 10 µL test substance, and 10 µL yeast solution (1:10 dilution in H_2_O, final OD600 of 0.1). Growth was monitored over 24 hours using an absorbance microplate reader (Bio Tek) maintained at 30°C, with measurements taken at 600 nm every 30 min after 15 s of orbital shaking. All measurements were performed in triplicate using independent overnight cultures conducted on different days. Growth was quantified as the area under the curve (AUC) over 24 hours and expressed as percent change compared to control setups (H_2_O instead of test substance) in the same medium. AUCs were determined for three replicates, and the mean change compared to controls is shown with SDs as error bars (22).

### Microbial species and growth conditions

The reference strain *C. albicans* SC5314 was obtained from Ningbo Mingzhou Biotechnology Co., Ltd (Ningbo, China). The strain was cultured in liquid YPD medium and incubated for 24 hours at 37°C under aerobic conditions.

Three *Lactobacillus* species were isolated from the vagina of healthy women: *L. crispatus*, *L. johnsonii*, and *L. gasseri*. For the experiments, *Lactobacillus* colonies were inoculated into 15 mL of liquid MRS (Hopebiol, China) and incubated at 37°C under anaerobic conditions for 24 hours. Microorganisms in the exponential growth phase were utilized in each experiment.

### Preparation of CFSs from *Lactobacillus* species and pH evaluation

The three *Lactobacillus* species were cultured in 15 mL of MRS liquid medium for 24 hours at 37°C under anaerobic conditions. CFSs from the *Lactobacillus* cultures were obtained by centrifuging the bacterial suspensions at 4,000 rpm for 15 minutes at 4°C, followed by collecting the supernatants and filtration through 0.20 µm syringe filters (Corning Incorporated, Germany). To ensure the absence of bacterial contamination in the CFS, 1 mL of each supernatant was incubated at 37°C for 24 hours, and turbidity was assessed post-incubation. The pH of each CFS was measured by a pH meter (Hanna Instruments, Italy). The control samples consisted of sterile MRS medium and acidified MRS medium (MRS-HCl). The obtained CFS were stored at −80°C until further use.

### Effect of CFS on *C. albicans* growth and activity

*C. albicans* was used at the concentration of 1 × 10^6^ CFU/mL. The ratio of *C. albicans* to CFS was maintained at 1:1. One hundred microliters of *C. albicans* suspended in YPD broth were mixed with 100 µL of various CFS, MRS, or acidified MRS at pH 4.8 (MRS-HCl). This mixture was incubated at 30°C for 24 hours in a 96-well microplate (Costar 3595, Corning, USA). The growth of *C. albicans* was quantified by measuring the optical density (OD) at 600 nm using a spectrophotometer (Sunrise, Tecan, Switzerland). Cell counts were determined using a hemocytometer, with dead cells excluded through trypan blue vital staining. Data were presented as OD600 mean ± SD and mean CFU/mL ± SD (58).

### Effect of CFS on *C. albicans* hyphal formation

*C. albicans* was used at the concentration of 4 × 10^6^ CFU/mL, and the ratio of *C. albicans* to CFS was 3:2. *C. albicans* was evaluated for hyphal formation in a YPD medium supplemented with 10% fetal bovine serum (Gibco, USA). In 96-well plates, 120 µL of *C. albicans* was combined with 80 µL of various CFS, MRS, or MRS-HCL and then incubated at 37°C for 1.5 hours. After incubation, 10 µL of each sample was recovered and placed on a glass microscope slide. Yeast cells and hyphal structures were counted in four fields for each experimental condition using an optical microscope (Nikon Eclipse 80i, Nikon Corporation, Japan). Data were presented as mean ± SD of hyphae percentage.

An overview of the specific quantities of the *Lactobacillus* CFS, *C. albicans*, and incubation periods used in each experiment is provided in Table 1.

**TABLE 1:** An overview of the exact number of the *Lactobacillus* CFS, *C. albicans*, and incubation periods used in each experiment

Experiment	Format incubation	CFU of C.a./mL (volume)	CFS of *Lactobacillus* volume	Incubation period
Growth assay	96-well plate	1 × 10^6^ CFU/mL (100 µL)	100 µL	24 hours
Viability assay	96-well plate	1 × 10^6^ CFU/mL (100 µL)	100 µL	24 hours
Hyphal formation assay	96-well plate	4 × 10^6^ CFU/mL (120 µL)	80 µL	1.5 hours

### Microbiome profiling

Subjects for this study were enrolled from April 2023 to May 2024 at the Reproductive Health Clinic of Hospital for Reproductive Medicine Affiliated to Shandong University. A total of 40 subjects aged 22–40 years were recruited, including 20 patients diagnosed with VVC and 20 healthy women. VVC was diagnosed by the identification of budding yeasts, hyphae, or pseudo-hyphae in a wet preparation (saline, 10% KOH) of vaginal discharge (59). The exclusion criteria were (i) pregnancy, breastfeeding, or menstruation; (ii) diagnosed with other urinary system diseases or systemic diseases; (iii) engaged in sexual intercourse, vaginal procedures, or douching within the last three days; (iv) history of hormonal treatment within the last 3 months; or (v) use of systemic or topical antibiotics or probiotics within the last month. Vaginal swabs were collected by inserting a sterile swab into one-third of the lateral wall of the subject’s vagina and rubbing the vaginal wall for 10–15 seconds. Two swabs were collected each time, and the swabs were immediately placed in a sterile, anaerobically sealed test tube filled with saline. One was then immediately processed for bacterial isolation, while the other, intended for metagenomic sequencing, was stored at −80°C until further analysis. Metagenomic sequencing was conducted by Novogene Co., Ltd. in Beijing, China (extended details are provided in Supplementary material Metagenomics sequencing). Species analysis was conducted using Kraken 2.1.2 (60).

### Statistics

All model improvements and analyses were conducted using COBRApy (v0.17.1) in Python 3.7, the academic version of IBM CPLEX solver (v12.9.0.0), R (v4.3.1), and GraphPad Prism 9.3.0 software (San Diego, CA, USA). LEfSe was used for differential analysis. The Spearman test was used for correlation analysis. Normally distributed experimental results, as determined by the Shapiro−Wilk normality test, were analyzed using a one-way analysis of variance (ANOVA) test followed by Dunnett’s multiple-comparison test. Values of **, *P* < 0.01; ***, *P* < 0.001; ****, *P* < 0.0001; ns, no significance.

## Data Availability

The data supporting the findings of this study are available in the supplemental material. The Python code and metabolic models for simulating pairwise GEMs are available at https://github.com/Hu-tianqi/Candida-albicans-Lactobacillus--interaction. The raw sequencing data have been deposited into the National Omics Data Encyclopedia (NODE; https://www.biosino.org/node/index) with the accession number OEP00005670.

## SUPPLEMENTAL MATERIAL

File S1 spectrum.02984-24-s0001.html MEMOTE report of *Candida albicans* metabolic model.

Figure S1 spectrum.02984-24-s0002.pdf *Candida albicans* metabolic reaction flux analysis with inhibiting *Lactobacillus* and KEGG pathway annotation.

Supplementary material spectrum.02984-24-s0003.docx Legends for supplementary figures and tables; extended details for model improvement and simulation, metagenomics sequencing.

Table S1 spectrum.02984-24-s0004.xlsx All *in silico* prediction results for individual and pairwise flux balance analysis experiments.

Table S2 spectrum.02984-24-s0005.xlsx In silico predictions for exchange reaction fluxes.

Table S3 spectrum.02984-24-s0006.xlsx Refinements for *Candida albicans* genome-scale metabolic model.

Table S4 spectrum.02984-24-s0007.xlsx Information for *Candida albicans* genome-scale metabolic model (comprehensive list including all reactions and metabolites).

Table S5 spectrum.02984-24-s0008.xlsx Selected *Lactobacillus* and metabolic models.

Table S6 spectrum.02984-24-s0009.xlsx General vaginal media composition and definition.

ASM does not own the copyrights to Supplemental Material that may be linked to, or accessed through, an article. The authors have granted ASM a non-exclusive, world-wide license to publish the Supplemental Material files. Please contact the corresponding author directly for reuse.
