# Supplementary material for: Genome-scale metabolic modeling reveals specific vaginal Lactobacillus strains and their metabolites as key inhibitors of Candida albicans
Source: Microbiol Spectr. 2025 Apr 16;13(6):e02984-24. doi: 10.1128/spectrum.02984-24 (PMC12186704; doi:10.1128/spectrum.02984-24)
Supplement: Figure S1 — Candida albicans metabolic reaction flux analysis with inhibiting Lactobacillus and KEGG pathway annotation. [file spectrum.02984-24-s0002.pdf]

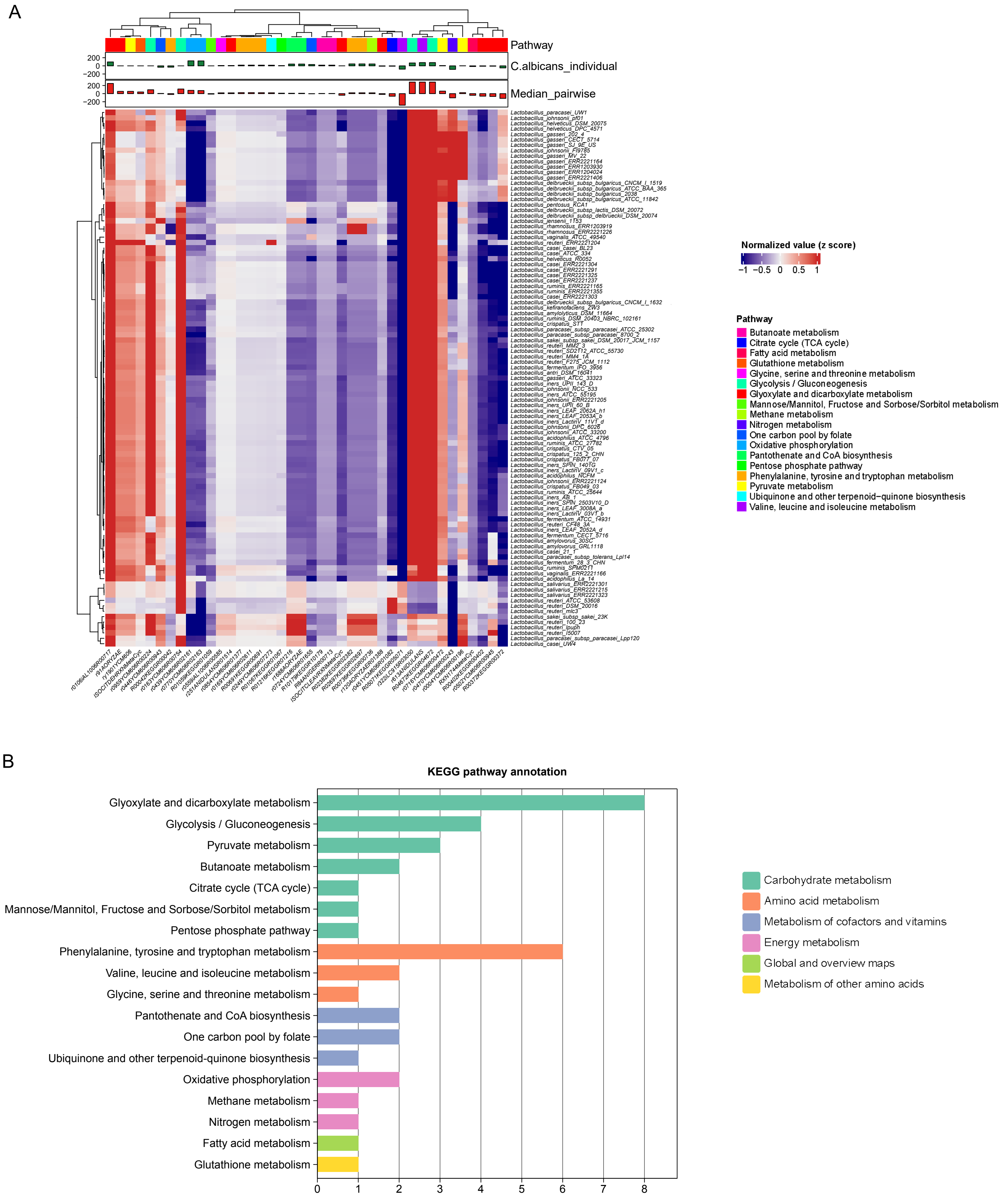

Supplementary Figure S1. **A** Metabolic reactions of *C. albicans* with the most substantially differing flux rates of *C. albicans* individual growth or paired with inhibiting *Lactobacillus*. Top: The flux of *C. albicans* when grown individually and the median flux of *C. albicans* when paired with inhibiting *Lactobacillus*. **B** KEGG pathway annotation of metabolic pathways.
