# Supplementary material for: Genome-scale metabolic modeling reveals specific vaginal Lactobacillus strains and their metabolites as key inhibitors of Candida albicans
Source: Microbiol Spectr. 2025 Apr 16;13(6):e02984-24. doi: 10.1128/spectrum.02984-24 (PMC12186704; doi:10.1128/spectrum.02984-24)
Supplement: Supplementary material — Legends for supplementary figures and tables; extended details for model improvement and simulation, metagenomics sequencing. [file spectrum.02984-24-s0003.docx]

**Supplementary Material**

Legends for separate supplementary figures and tables; extended details for model improvement and simulation, metagenomics sequencing

**Separate supplementary figures and tables - Legends**

Supplementary Figure S1: *Candida albicans* metabolic reactions flux analysis with inhibiting *Lactobacillus* and KEGG pathway annotation

Supplementary File S1: MEMOTE report of *Candida albicans* metabolic model

Supplementary Table S1: All in silico prediction results for individual and pairwise Flux Balance Analysis experiments

Supplementary Table S2: In silico predictions for exchange reaction fluxes

Supplementary Table S3: Refinements for *Candida albicans* genome-scale metabolic model

Supplementary Table S4: Information for *Candida albicans* genome-scale metabolic model (comprehensive list including all reactions and metabolites)

Supplementary Table S5: Selected *Lactobacillus* and metabolic models

Supplementary Table S6: General vaginal media composition and definition

**Pairwise simulations**

An organism was considered to be a promoter or inhibitor if the growth rate of the paired organism increased or decreased by at least 10% in the paired simulation compared to the individual growth rate. Six different ecological interaction types of mutualism, commensalism, amensalism, neutralism, parasitism, and competition were assigned based on whether *Lactobacillus* promoted, inhibited or had neutral effects on *C. albicans* and vice versa.

To identify *C. albicans* reactions with pronounced flux differences in *C. albicans* grown individually and paired with inhibiting *Lactobacillus*, reactions were first filtered to show at least 80% consistent flux direction for each group of paired simulations. The differences in median flux for each selected reaction in the *C. albicans* paired with the inhibiting bacteria, compared to the flux of the same reactions in *C. albicans* grown individually, were calculated to identify different reactions. These differences were then visualized as a heat map to highlight the changes in flux associated with the presence of the inhibiting *Lactobacillus*. Similarly, to analyze the reactions of *C. albicans* under paired simulations with both inhibiting and promoting *Lactobacillus*, we again filtered the reactions to maintain at least 80% consistency in flux direction for each group. We determined the flux differences by calculating the median flux values for each selected reaction across all *C. albicans* models in both conditions (paired with inhibiting versus promoting bacteria).

## Metagenomics sequencing

1. Experimental Procedure

A total amount of 0.2 μg DNA per sample was used as input material for the DNA library preparations. Briefly, genomic DNA sample was fragmented by Covaris LE220R-plus (Covaris, USA) to a size of 350 bp. Then DNA fragments were endpolished, A-tailed, and ligated with the full-length adapter for Illumina sequencing, followed by further PCR amplification. PCR products were purified by AMPure XPsystem (Beckman Coulter, Beverly, USA). Subsequently, library quality was assessedon the Agilent 5400 system (AATI) and quantified by real-time PCR (1.5 nM). The qualified libraries were pooled and sequenced on Illumina platforms with PE150 strategy in Novogene Bioinformatics Technology Co., Ltd (Beijing,China), according to effective library concentration and the data amount required.

2. Data quality control

2.1 Raw data

The original fluorescence image files are transformed to short reads (Raw data) by base calling and these short reads are recorded in FASTQ format, which contains sequence information and corresponding sequencing quality information.

2.2 Evaluation of data (Data quality control)

Sequence artifacts, including reads containing adapter contamination, low-quality nucleotides and unrecognizable nucleotide (N), undoubtedly set the barrier for the subsequent reliable bioinformatics analysis. Hence quality control is an essential step and applied to guarantee the meaningful downstream analysis. we used Fastp to perform basic statistics on the quality of the raw reads. The steps of data processing were as follows:

(1) Discard a pair of reads if either one of them contains adapter contamination(>10 nucleotides aligned to the adapter, allowing ≤ 10% mismatches);

(2) Discard a pair of reads if more than 10% of bases are uncertain in either one of the reads;

(3) Discard a pair of reads if the proportion of low quality (Phredquality<5) bases is over 50% in either one of the reads.
